# Clinical heterogeneity in a family with flail arm syndrome and review of *hnRNPA1*‐related spectrum

**DOI:** 10.1002/acn3.51682

**Published:** 2022-10-31

**Authors:** Xiaochen Han, Feixia Zhan, Yuxin Yao, Li Cao, Jianguo Liu, Sheng Yao

**Affiliations:** ^1^ Department of Neurology The Sixth Medical Center of PLA General Hospital Beijing 100048 China; ^2^ Department of Neurology Shanghai Sixth People's Hospital Affiliated to Shanghai Jiao Tong University School of Medicine 200233 Shanghai China

## Abstract

**Objective:**

Flail arm syndrome (FAS) is one of the atypical subtypes of amyotrophic lateral sclerosis (ALS). Mutations in *hnRNPA1* encoding heterogeneous nuclear ribonucleoprotein (hnRNP) A1 are a rare genetic cause of ALS. Herein, marked clinical heterogeneity of FAS in a pedigree with a known *hnRNPA1* variant was described to raise early awareness of the ALS variant. Furtherly, a literature review of the *hnRNPA1*‐related spectrum was made to summarize the clinical and genetic characteristics.

**Methods:**

Detailed clinical evaluation, muscle pathology, and whole‐exome sequencing were performed. The sequence and co‐segregation of the mutation among the family members were confirmed by Sanger sequencing.

**Results:**

The great clinical variability was found in a FAS pedigree. Muscle pathology revealed a cluster distribution of angulated or rounded atrophic fibers, accompanied by significant multi‐nucleus aggregation. Immunohistochemical staining showed that mutant hnRNPA1 proteins accumulated in muscle fiber cytoplasm. Exome sequencing identified a documented variant in *hnRNPA1* gene c.1018C > T (p.P340S), which co‐segregated with disease in the family. Besides, highly phenotypic heterogeneity was also found in other *hnRNPA1*‐related diseases.

**Interpretation:**

We described a Chinese pedigree with *hnRNPA1*‐related FAS, which showed significant clinical variability among the intrafamilial members. FAS is a relatively milder variant of ALS, due to the highly heterogeneous clinical spectrum, early observation is of paramount importance. In addition, the highly phenotypic heterogeneity and molecular genetic mechanism of the *hnRNPA1*‐related spectrum are still beyond fully understood. Further, the detailed molecular mechanism underlying the clinical diversity is warranted to be explored.

## Introduction

Amyotrophic lateral sclerosis (ALS) has been described as a progressive and ultimately fatal neurodegenerative disease characterized by selective degeneration of cortical, brainstem, and spinal cord motor neurons.^1^ Based on different sites of onset, the extent of upper and lower motor neuron dysfunction, and the rate of progression, the disease shows significant phenotypic variability. The main phenotypic categories include classic limb‐onset ALS, bulbar‐onset ALS, primary lateral sclerosis, progressive muscular atrophy, and progressive bulbar palsy, all of which are closely associated with both diagnostic and prognostic significance.^2^, ^3^ Moreover, several atypical variants of motor neuron disease‐ALS have also been reported, including flail limb syndrome (flail‐arm and flail‐leg syndrome), facial‐onset sensory and motor neuronopathy, finger extension weakness and downbeat nystagmus, and juvenile ALS.^4^, ^5^ Flail arm syndrome (FAS), first described in 1886, was also referred to as Vulpian‐Bernhardt syndrome, man‐in‐the‐barrel syndrome, hanging‐arm syndrome, or brachial amyotrophic diplegia. The condition is characterized by predominantly lower motor neuron involvement with proximal, progressive weakness and wasting of the upper limbs, without significant functional effects on other regions in the early stage.^5^, ^6^, ^7^ Although patients with FAS presented with similar electrophysiological and pathological features as in typical ALS, it is commonly asymmetric at symptom‐onset, then being classically bilateral after a variable period of months to years.^8^, ^9^, ^10^, ^11^ The syndrome was associated with a slower progression and better prognosis than typical ALS or progressive muscular atrophy,^4^, ^12^ indicating that FAS is a relatively milder variant of ALS, and early awareness of the disease is crucial to prompt accurate diagnosis and proper management.

The causes of ALS are still largely unknown, with 90% of cases being sporadic and the remaining 10% have a genetic component.^13^ So far, mutations in genes that encode RNA‐binding proteins (RBPs), including FUS, TDP‐43, hnRNPA1, hnRNPA2B1, MATRIN3, TAF15, and TIA1, are being recognized as causal drivers or associated with multiple neurodegenerative diseases, such as ALS, multisystem proteinopathy, and frontotemporal dementia.^14^, ^15^ There are many structural similarities between these RBPs, like the RNA recognition motifs, a glycine‐rich region, and multiple C‐terminal arginine‐glycine–glycine (RGG) domains. In addition, these RBPs share functional commonalities. They are predominantly nuclear, multifunctional proteins that are widely expressed and implicated in a broad range of cellular processes.^15^ An increasing number of mutations identified in RBPs have been shown to interrupt stress granule dynamics and increase aggregation, suggesting that the dysfunctional RBPs and dysregulated RNA homeostasis are involved in the pathogenesis of neurodegeneration.^16^ Recently, pathogenic *hnRNPA1* variants have been reported to cause FAS through dysregulated polymerization and adverse consequences for RNA metabolism,^17^ while the underlying mechanism associated with the milder and variable phenotype of FAS remains unclear. Although FAS has been recognized since the late 19th century, it remains inadequately characterized. Due to the heterogeneous phenotypes and similar clinical mimics, the diagnosis may not only be clinically missed in the early stages but worse, the patient may be wrongly labeled. Herein, marked clinical heterogeneity of FAS in a pedigree with a known *hnRNPA1* mutation was described, and a detailed review of the *hnRNPA1*‐related spectrum was further made to summarize the clinical and genetic characteristics, enhance the early recognition, and shed light on the pathogenic study for these diseases.

## Material and Methods

### Patients

An extensive clinical evaluation was performed on a 42‐year‐old Chinese female. Clinical diagnosis of definite or probable ALS based on the revised El Escorial criteria^18^ or FAS based on previous description^19^ by at least two neurologists. Some family members were also clinically examined.

### Ethical approval

This study was approved by the ethics committee of the Sixth Medical Center of PLA General Hospital, Beijing, China. Written informed consent, which also included the consent for the publication of medical information, was obtained from the proband and her family members.

### Muscle pathology

An open muscle biopsy was performed on the right biceps brachii muscle of the proband according to a standard procedure. The tissue was frozen and then cut into 8‐*μ*m sections. For histological examination, serial frozen sections were stained by conventional histochemical techniques, including hematoxylin & eosin (H&E), modified Gomori trichrome, Oil Red O, periodic acid Schiff, succinate dehydrogenase, nicotinamide adenine dinucleotide tetrazolium reductase, cytochrome c oxidase, nonspecific esterase, and muscle fiber ATPase at varying pH levels. For immunohistochemical staining, sections were stained with the following primary antibodies: dystrophin, sarcoglycans, dysferlin, major histocompatibility complex class‐I (MHC‐I), C5b‐9, CD3, CD4, CD8, CD20, CD68, and hnRNPA1 (bs‐17330R, Bioss). A patient with asymptomatic hyperCKemia was set as a control to investigate the hnRNPA1‐related pathological changes.

### Genetic analyses

Genomic DNA was extracted from peripheral blood using the standardized phenol/chloroform extraction protocol. Exome sequencing was performed using Agilent SureSelect v6 reagents for capturing exons and Illumina HiSeq X Ten platform for sequencing. The frequency and predicted pathogenesis of the detected variants were evaluated with various population databases and software, including 200 in‐house ethnically matched healthy controls, the 1000 Genomes Project (http://www.internationalgenome.org), the Single Nucleotide Polymorphism Database (http://www.ncbi.nlm.nih.gov/projects/SNP), the gnomAD database, the Human Gene Mutation Database (http:www.hgmd/.cf.ac.uk/ac/index.php), MutationTaster (http://www.mutationtaster.org), PolyPhen‐2 (http://genetics.bwh.harvard.edu/pph2), and SIFT (http://sift.jcvi.org). Then the pathogenic of the variant was interpreted and classified following the American College of Medical Genetics and Genomics (ACMG) Standards and Guidelines.^20^ Sanger sequence and co‐segregation analysis were further confirmed among family members.

## Results

### Clinical characteristics of the FAS family

**Figure 1:**
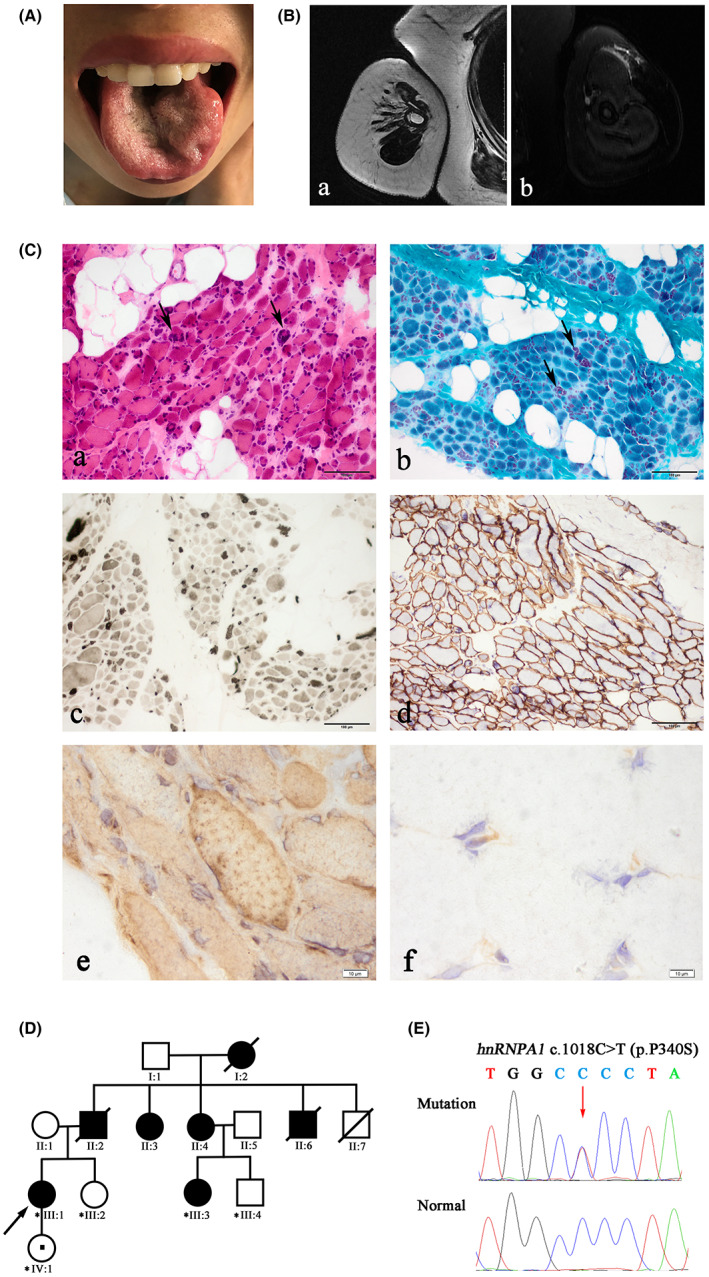
Clinical sign, muscle pathology, and genetic characterizations. (A) The bilateral atrophic tongue muscle of the proband. (B) The muscle MRI of the right upper limb of the proband. The coronal view showed apparent atrophy with infiltration of fatty tissue on the T1‐weighted imaging (a) and no tissue edema on the STIR imaging (b). (C) Muscle pathology of the proband revealed a cluster distribution of angulated or rounded atrophic fibers with significant multi‐nucleus aggregation (arrow) and infiltration of fatty tissue by H&E (a) and modified Gomori trichrome (b) X 400. Fiber type disproportion with larger type 2 fibers than type 1 on ATPase staining pH 4.5 (c). Immunohistochemical staining of dystrophin was normal (d). Immunohistochemical staining of hnRNPA1 showed that mutant proteins accumulated in muscle fiber cytoplasm (e), compared with an exclusively nuclear localization in a control case with asymptomatic hyperCKemia (f) X 1000. (D) The family pedigree is shown. Filled‐in symbols indicate individuals with muscle weakness or tongue atrophy. Empty symbols indicate unaffected individuals without any medical history or complaints of muscle weakness. Arrow = proband. Individual IV:1 is the asymptomatic mutation carrier. (E) Sanger sequencing confirmed the heterozygous *hnRNPA1* mutation (c.1018C > T) (NM_031157). Asterisks indicate individuals whose DNA was available for the Sanger sequence.

The index patient (individual III:1) was a 42‐year‐old Chinese female from a nonconsanguineous family referred to our neurology department with a 9‐year history of slowly progressive weakness and wasting of her right arm, accompanied by the involuntary beating of facial and neck muscles. As the disease progressed, she presented with gradually aggravated slurred speech, weakness in chewing, and facial cramps at the age of 39. About 2 days before admission, she had difficulty swallowing, causing suffocating to wake up at night with severe coughing and drooling. Positive family history was obtained with multiple family members affected. Neurological examination revealed that bulging and whistling cannot be conducted due to bilateral temporomandibular muscle weakness, along with weak turning and shrugging on the right side. It also could be found involuntary twitching of the perioral and neck muscles, and tremors of the bilateral atrophic tongue muscle (Fig. 1A). The muscle strength of the atrophic right upper arm was decreased, while the distal was normal. The patient also showed positive upper motor neuron signs, including rectus abdominis reflex, bilateral Rosslimo sign, and ankle clonus. However, no evident weakness or spasticity in the lower limbs was observed. The sensation was intact. Bilateral Hoffmann and Babinski signs were not elicited. Laboratory tests of serum and cerebrospinal fluid, and the cervical and brain MRI were normal. The muscle MRI of the right upper limb showed apparent atrophy with infiltration of interstitial fatty tissue (Fig. 1B‐a). Needle electromyogram showed neurogenic abnormality in right biceps brachii, right interosseous, right sternocleidomastoid, and right thoracic 10 paraspinous with loss of motor unit potential (MUP), and decreased recruitment and reinnervation potential (giant spike and polyphasic MUP).

The family pedigree is shown in Figure 1D. The proband's father (individual II:2) was onset at the age of 30 with a 6‐year history of progressive slurred speech, weakness, and wasting of bilateral upper limbs. He developed dysphagia as the disease progressed and died of respiratory failure at the age of 36. Patient II:3 is a 58‐year‐old woman. She manifested weakness in the proximal upper limbs at the age of 30, and dysarthria occurred during disease progression. Currently, she can take care of herself in her daily life. Patient II:4, aged 53 years, had weakness and wasting of proximal upper limbs at the age of 44, while there were no symptoms of dysarthria and dysphagia presently. Patient III:3 is a 30‐year‐old female who suffered from vague speech and diet choking at 27 with no complaint of weakness or muscular twitching. Her physical examination observed lingual muscle atrophy and fibrillation. Muscle strength and tension of all limbs were normal, and there was no muscular atrophy. Bilateral Hoffmann or Babinski signs were not elicited. Patient II:6 was onset at the age of 35 with progressive weakness of both upper limbs. He died of dysphagia and increased slavering at the age of 40. The clinical features of all affected family members are summarized in Table 1.

**Table 1:** The clinical features of all affected family members.

Individual No.	Sex	AAO (y)	Disease course (y)	Initial symptoms	Disease progression	*hnRNPA1* mutation
III:1	F	33	9	Weakness and wasting of the right arm, accompanied by the involuntary beating of facial and neck muscles.	Aggravated slurred speech, weakness in chewing, difficulty swallowing, and facial cramps.	c.1018C > T (p.P340S)
II:2	M	30	6	Progressive slurred speech, weakness, and wasting of bilateral upper limbs.	Dysphagia developed, died of respiratory failure.	NP
II:3	F	30	28	Weakness in the proximal upper limbs.	Dysphagia developed, she could take care of herself in the daily life at present.	NP
II:4	F	44	9	Weakness and wasting of proximal upper limbs.	No obvious progress so far.	NP
II:6	M	35	5	Weakness of both upper limbs.	Died of dysphagia and increased slavering.	NP
III:3	F	27	3	Vague speech and diet choking.	Atrophy and fibrillation of the lingual muscle.	c.1018C > T (p.P340S)

F = female; M = male; AAO = age at onset; y = year; NP, not performed.

### Myopathological findings

Muscle pathology of the proband showed a cluster distribution of grouped angulated or rounded atrophic fibers, accompanied by significant multi‐nucleus aggregation, and infiltration of fatty tissue (Fig. 1C‐a, b). A tendency toward type 2 fiber predominance and fiber‐type disproportion was found on ATPase staining (pH 4.5) (Fig. 1C‐c). There was no muscle fiber necrosis, regeneration, division, and inclusion body. No abnormalities in the immunohistochemical staining of dystrophin (Fig. 1C‐d), sarcoglycans, dysferlin, and MHC‐I. However, mutant hnRNPA1 proteins accumulated in the cytoplasm of muscle fibers (Fig. 1C‐e), compared with an exclusively nuclear localization in the control case with asymptomatic hyperkalemia (Fig. 1C‐f).

### Genetic findings

Exome sequencing identified a documented heterozygous *hnRNPA1* (NM_031157) variant c.1018C > T (p.P340S) (Fig. 1 E), which was previously reported by Liu's study in an unrelated FAS family.^21^ The mutation was not found in 1000 Genomes Project, dbSNP, gnomAD, as well as 200 in‐house ethnically matched healthy controls. The silico analysis of this variant was predicted to be disease‐causing by Mutationtaster (probability score:1.000), and possibly damaging by PolyPhen2 (probability score:0.999), while tolerable by SIFT (probability score:0.051). Sanger sequencing showed that patient III:3 and the proband's asymptomatic daughter (individual IV:1) shared the variant, while unaffected individuals III:2 and III:4 did not carry the mutation. The co‐segregation analysis of other affected members failed to be conducted due to the unavailability of related DNA samples. Pathogenicity assessment according to the ACMG revealed that the mutation is pathogenic.

## Discussion

ALS is a highly heterogeneous entity, phenotypic variability goes far beyond the age and site of onset, rate of progression, familial occurrence, type of motor neuron involvement, and extent of extramotor involvement.^22^ Neuropathologically, the disease is characterized by the loss of upper motor neurons (UMN) and lower motor neurons (LMN), as well as the variable distribution of motor neuronal cytoplasmic inclusions.^23^ Although there are several known genetic risks for ALS, about 90% of cases do not have a definite genetic cause.^24^ Besides, the complex genetics and pathophysiology are not fully understood. Considering the variable neuropathological features and the complicated etiologies of ALS, the true heterogeneity is likely to be even larger. Despite a common rapidly progressive course of the classical motor neuron disease (MND), atypical presentations of ALS with a slow progression and deficits early confined to a focal anatomical region (limb, bulbar, or respiratory) or only to one motor type (UMN or LMN) have been widely described,^5^ indicating that clinicians should attach importance to the heterogeneous and expanding spectrum of MND/ALS.

In the present study, we described a genetically confirmed FAS family with marked phenotypic heterogeneity. FAS is one of the atypical and mild variants of ALS, accounting for about 10%.^7^ It was reported that the mean age at symptom onset was 50–60 years, with a higher male‐to‐female ratio (4–10:1).^4^, ^19^, ^25^ The deltoid, supraspinatus, infraspinatus, sternocleidomastoid, and teres minor muscles of these patients are severely atrophied, resulting in a characteristic posture, that is, the shoulders are sunken, the upper arms, forearms, and hands are pronated.^6^ However, lower limbs or bulbar muscles were long‐term spared or slightly affected. The condition progresses slowly with diverse initial symptoms and asymmetric onset. Moreover, the affected area is limited to the neck for a relatively long time.^12^, ^25^ In our study, the mean onset age of affected members was 33.2 ± 5.5 years (range, 27 to 44 years), which was quite earlier than the previous study.^4^, ^25^ In addition, significant phenotypic variability was observed among family members with the same genotype, like the variable onset age, onset sites, as well as disease progression and severity. Besides, the proband's 20‐year‐old asymptomatic daughter carried the pathogenic variant with no symptoms or signs currently, which had never been described and needed continuous follow‐up. The great clinical heterogeneity among the interfamily members with identical genotypes points to the possibility of interaction among genetic, epigenetic, and environmental factors. In clinical practice, patients with FAS can be easily misdiagnosed due to the heterogeneous and atypical presentations in the early stage,^26^ suggesting the significance of a more detailed description of the clinical and electrophysiological pictures, as well as the early specific markers of FAS. When presenting with chronic progressive unilateral or bilateral upper limb weakness, and muscle atrophy, with or without bulbar symptoms, the differential diagnosis including FAS should be considered.

The pathogenesis of ALS remains largely unclear. The only reliably reproducible risk factors are increasing age, being male, or having a positive family history.^1^ Familial ALS is caused by multiple factors including mutations in several specific genes.^27^ Recently, mutations in several genes that encode RNA‐binding proteins (RBPs) have emerged as critical determinants of neurological diseases, especially ALS and multisystem proteinopathy (MSP).^15^, ^28^, ^29^ Human heterogeneous nuclear ribonucleoprotein (hnRNP) A1 is a member of a large class of RBPs that assemble with RNA to form ribonucleoproteins. hnRNPs are evolutionarily conserved and are primarily localized to the nucleus, where they are involved in a wide range of RNA‐processing events including synthesis, transcription, splicing, stability, localization, translation, and decay.^30^, ^31^ In 2013, Kim et al. firstly described two families, one with MSP and another with ALS harboring the damaging *hnRNPA1* variants.^17^ The coding protein hnRNPA1 is widely expressed and shuttles continuously between the nucleus and cytoplasm. It contains two globular RNA recognition motifs for the specific binding with mRNA precursor in the N‐terminal, a C‐terminal prion‐like domain (PrLD), containing a cluster of RGG repeats that facilitate non‐specific RNA binding and protein interactions, and a proline‐tyrosine nuclear localization signal (PY‐NLS) that serves as both nuclear localization and export.^15^, ^28^, ^32^ As previous studies shown, the variant p.P340S in the PrLD region of hnRNPA1 altered stress granule dynamics through cytoplasmic mislocalization and accelerated fibrillization,^17^, ^21^, ^33^, ^34^ which confirmed the underlying cause of our patient. Besides, hnRNPA1 has been reported to interact with many other ALS‐linked proteins, such as TDP‐43, which may cause disease through affected interactions between proteins.^15^, ^35^, ^36^ What is more, hnRNPA1 was shown to autoregulate its alternative splicing through negatively regulating mRNA levels, which was essential for maintaining the protein at a non‐cytotoxic level.^37^ Although the mechanisms of pathogenicity of *hnRNPA1* variants are not well studied, toxic misfolded protein, reduced normal function, and alterations in overall protein levels or a combination might be implicated.

Mutations in *hnRNPA1*, though relatively rare,^38^, ^39^ also show marked clinical heterogeneity. Up to now, there were 12 *hnRNPA1* variants have been reported in a total of 35 patients from 13 families of different ethnic groups, among which manifested with distal myopathy, distal hereditary motor neuropathy, and different phenotypes of the MSP spectrum including ALS, inclusion body myopathy with early‐onset Paget disease.^17^, ^21^, ^33^, ^34^, ^40^, ^41^, ^42^ The detailed clinical presentations of these patients are shown in Table S1. The ratio of male to female was 1.92:1, and the age of onset spanned a wide range, which could range from the first decade to the late 60 s, while most of them were mainly concentrated between 35 and 45 years old. Muscle weakness and inconvenient walking were the common initial symptoms, and the progression of the disease was slow or moderate with intrafamilial variability, none of these patients showed any intellectual deficit. Almost all patients manifested with myopathy showed moderate to severe myopathic‐dystrophic features combined with numerous rimmed vacuoles and eosinophilic cytoplasmic inclusions on pathological examination. Due to the limited cases, we could not reach a definite conclusion on the association between various phenotypes and the *hnRNPA1* genotype. In addition, single nucleotide variants in *hnRNPA1* have also been demonstrated to be implicated in the pathogenesis of multiple sclerosis.^43^ The wide inter‐ and intra‐familial phenotypic spectra of *hnRNPA1* mutations, and the loose genotype–phenotype correlation suggest the involvement of secondary epigenetic or environmental factors in the clinical phenotype. Nevertheless, the highly phenotypic heterogeneity and the molecular genetic mechanism of these diseases are still beyond clearly explained. It is noteworthy that the potential common pathogenic role of the RBPs involved in neurodegenerative diseases remains to be further elucidated, which may shed light on the pathogenic studies and achieve a breakthrough in therapeutic approaches.

In conclusion, we described a Chinese pedigree with *hnRNPA1*‐related FAS, which showed high clinical heterogeneity among the intra‐familial members. FAS is a relatively milder variant of ALS with a slower progression and better prognosis, due to the atypical clinical spectra, early observation is crucial to prompt the proper intervention and facilitate appropriate counseling. Besides, we are still far from a comprehensive picture of the detailed molecular mechanism of the great clinical variability of the *hnRNPA1*‐related spectrum. Identification of the underlying mechanisms of RBPs may hold promise for the development of therapies for a broad spectrum of neurodegenerative diseases.

## Conflict of Interest

The authors declare that they have no conflict of interest.

## Author Contributions

Material preparation, data collection, and analysis were performed by Xiaochen Han, Feixia Zhan, Yuxin Yao, Li Cao, Jianguo Liu, and Sheng Yao. The first draft of the manuscript was written by Xiaochen Han and Feixia Zhan. The manuscript for intellectual content was revised by Jianguo Liu and Sheng Yao. All authors read and approved the final manuscript.

## Supporting information


**Table S1.** The clinical presentations of all patients with the *hnRNPA1* variant.
