# Supplementary material for: Clinical heterogeneity in a family with flail arm syndrome and review of hnRNPA1‐related spectrum
Source: Ann Clin Transl Neurol. 2022 Oct 31;9(12):1910–7. doi: 10.1002/acn3.51682 (PMC9735363; doi:10.1002/acn3.51682)
Supplement: Supplementary file 1 — Table S1. The clinical presentations of all patients with the hnRNPA1 variant. [file ACN3-9-1910-s001.docx]

Supplemental Table 1. The clinical presentations of all patients with *hnRNPA1* variant

| **Researcher publication year** | **Individual** | **Ethnicity** | **Sex** | **AAO**  **(y)** | **Initial symptoms** | **Overall phenotypes** | **Disease course**  **(y)** | **Pathology** | ***hnRNPA1* mutation** |
| --- | --- | --- | --- | --- | --- | --- | --- | --- | --- |
| Hackman et al. 2021 | Case1 (II:7) | Finnish | F | 32-45 | Hand weakness and stumbling on the feet | Distal myopathy | N.A | Not performed | 160bp deletion spanning exon10  (p.G56Nfs*4) |
|  | Case2 (II:10) |  | F |  | Walking difficulty | Distal myopathy | 18 | Not performed |  |
|  | Case3 (III:3) |  | F |  | Hand weakness and stumbling on the feet | Distal myopathy | N.A | Not performed |  |
|  | Case4 (III:5) |  | M |  | Hand weakness and stumbling on the feet | Distal myopathy | N.A | Not performed |  |
|  | Case5 (III:6) |  | M |  | Hand weakness and stumbling on the feet | Distal myopathy | Died at 66 | myopathology with RVs, eosinophilic protein inclusions. |  |
|  | Case6 (IV:6) |  | M |  | Hand weakness and stumbling on the feet | Distal myopathy | N.A | Not performed. |  |
|  | Case7 (IV:7) |  | M |  | Hand weakness and stumbling on the feet | Distal myopathy | N.A | Advanced myopathology with RVs, eosinophilic protein inclusions. |  |
|  | Case8 (IV:11) |  | M |  | Foot drop, atrophy of small hand muscles | Distal myopathy | 14 | Minor fiber size variation and internal nuclei, very few RVs. |  |
| Beijer et al. 2021 | Case9 (A:II:1) | Polish | M | 15 | Wasting of hand intrinsics | dHMN | 28 | Mild axonal neuropathy with demyelination. | c.908-2A>G (p.G304Nfs*3) |
|  | Case10 (B:II:1) | Moroccan | F | 22 | Paresis of left hand | MND | 22 | Not performed. | c.862/1018C>G (p.P288/340A) |
|  | Case11 (B:II:2) |  | F | 43 | Dysphagia and dysarthria. | MND | 2 | Not performed. |  |
|  | Case12 (C:II:2) | Dutch | F | 9 | Hand function difficulties, facial weakness, fatigue. | Distal myopathy | 35 | Angular fibers, no type grouping. | c.961T>G (p.*321Eext*6) |
|  | Case13 (C:III:1) |  | M | 8 | Right-sided foot drop. | Distal myopathy | 6 | Slight clustering type 1 fibers. |  |
|  | Case14 (D:II:1) | India | F | 22 | Hand weakness while writing. | Distal myopathy | 13 | Myofibrillar disorganization, RVs. | 500bp deletion encompassing exon 9 (p.G304Nfs*3) |
|  | Case15 (E:II:2) | American | M | 36 | Weakness right foot, frequent falls. | Distal myopathy | 12 | Chronic myopathy with RVs. | c.785A>T  (p.D262V) |
|  | Case16 (F:I:1) | Belgian | M | 12 | Wasting of hand intrinsics, weakness lower limbs. | Distal myopathy | 52 | Type 1 atrophy, RVs, and centralized nuclei type 2 fibers. | c.961T>C (p.*321Qext*6) |
| Naruse et al. 2018 | Case17 (C:II:4) | Japanese | M | 36 | Weakness and atrophy of upper limbs. | FALS | 20 | Not performed | c.862/1018C>G (p.P288/340A) |
|  | Case18 (C:III:4) |  | M | 27 | Weakness and atrophy of upper limbs. | FALS | 5 | Not performed |  |
| Liu et al. 2016 | Case19 (III:2) | Chinese | M | 35 | Wasting and weakness of neck and arms. | FAS | 5 | Not performed | c.862/1018C>T (p.P288/340S) |
|  | Case20 (II:1) |  | M | 68 | Slight tremor in left hand and atrophy of thenar. | FAS | unknown | Not performed |  |
|  | Case21 (II:3) |  | F | 35 | Weakness in the proximal right arm. | FAS | 31 | Not performed |  |
|  | Case22 (II:5) |  | F | 32 | Inability of lifting head and proximal arms. | FAS | 32 | Not performed |  |
|  | Case23 (II:8) |  | M | 30 | Inability of lifting head and proximal arms. | FAS | 30 | Not performed |  |
| Izumi et al. 2015 | Case24(F1:III:1) | Japanese | M | 41 | Weakness and atrophy in the scapular, proximal and distal lower limbs. | IBM | 8 | RVs in atrophic fibers along with a chronic myopathic change | c.940G>A  (p.D314N) |
|  | Case25(F1:III:2) |  | M | 45 | Weakness in thighs, walking difficulty | IBM | 10 |  |  |
|  | Case26(F2:IV:1) |  | M | 49 | Walking difficulty | IBM | 13 |  |  |
|  | Case27(F2:IV:2) |  | M | 40 | Walking difficulty | IBM | 7 |  |  |
| Kim et al. 2013 | Case28 (b:IV:5) | German | M | 35 | Weakness beginning in the lower proximal extremities, and elevated serum creatine kinase | Myopathy and PDB | 23 | Myopathic, no RVs or inclusion bodies | c.785/941A>T  (p.D262/314V) |
|  | Case29 (b:IV:7) |  | F | 43 |  | Myopathy and PDB | 11 | Not performed |  |
|  | Case30 (b:IV:8) |  | M | 43 |  | Myopathy and PDB | 10 | RVs, inclusion bodies |  |
|  | Case31 (b:IV:9) |  | M | 39 |  | Myopathy and PDB | 9 | RVs, inclusion bodies |  |
|  | Case32(b:IV:10) |  | M | 42 |  | Myopathy and PDB | 6 | Not performed |  |
|  | Case33 (c:III:2) | N.A | F | N.A | N.A | ALS | N.A | Not performed | c.784/940G>A  (p.D262/314N) |
|  | Case34 (c:IV:1) |  | M | N.A | N.A | ALS | N.A | Not performed |  |
|  | Case35 (II:1) | N.A | F | N.A | N.A | Late-onset sporadic ALS | N.A | Not performed | c.800/956A>G  (p.N267/319S) |

F: female; M: male; AAO: age at onset; y: years; bp: base pair; dHMN: distal hereditary motor neuropathy; MND: motor neuron disease; FALS: familial amyotrophic lateral sclerosis; FAS: flail arm syndrome; IBM: isolated inclusion body myopathy; PDB: Paget's disease of bone; N.A: not available; RVs: rimmed vacuoles; ALS: amyotrophic lateral sclerosis.
